# Transient Spin Labeling of Plastics with Chlorine Dioxide

**DOI:** 10.1021/acsphyschemau.5c00140

**Published:** 2026-03-04

**Authors:** Bence G. Márkus, Sándor Kollarics, Kristóf Kály-Kullai, Bernadett Juhász, Dávid Beke, László Forró, Zoltán Noszticzius, Ferenc Simon

**Affiliations:** † Stavropoulos Center for Complex Quantum Matter, Department of Physics and Astronomy, 167055 University of Notre Dame, Notre Dame, Indiana 46556, United States; ‡ Department of Physics, Institute of Physics, 172285 Budapest University of Technology and Economics, Műegyetem Rkp. 3, Budapest H-1111, Hungary; § Institute for Solid State Physics and Optics, 162159 HUN-REN Wigner Research Centre for Physics, PO. Box 49, Budapest H-1525, Hungary; ∥ Kandó Kálmán Faculty of Electrical Engineering, Óbuda University, Tavaszmező U. 17, Budapest H-1084, Hungary

**Keywords:** chlorine dioxide (ClO_2_), polyethylene terephthalate (PET), spin labeling, electron spin resonance, spin Hamiltonian, plastic waste, environmental challenges

## Abstract

Plastic waste, one of the most critical problems for humankind, poses severe threats to ecosystems, wildlife, and human health. Tracing, quantifying, and identifying types of plastic waste are crucial to understanding its environmental pathways and developing targeted strategies for reduction, recycling, and remediation. To contribute to addressing this global issue, we investigated the spin-labeling capabilities of chlorine dioxide (ClO_2_) radicals introduced into poly­(ethylene terephthalate) and utilized electron spin resonance spectroscopy for detection. The technique is capable of identifying plastic species, as the unpaired electron of the radical molecule is strongly sensitive to its local environment through its coupling parameters. Temperature-dependent measurements revealed that the molecules are immobilized at low temperatures and exhibit well-resolved anisotropic and hyperfine spectra that are quantitatively described by a model spin Hamiltonian. Even above the melting point of water, certain degrees of freedom remain restricted as a result of the polymer matrix. Furthermore, employing a time-series measurement at room temperature enabled us to determine the diffusion coefficient of the molecule in the polymer.

## Graphical abstract

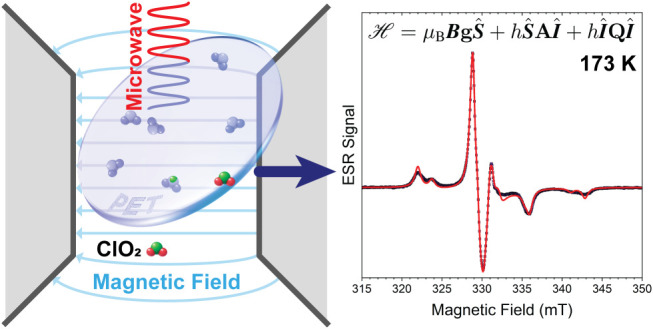

## 1 Introduction

The global accumulation of plastic waste represents one of the most pressing contemporary environmental challenges.^1,2^ Despite increasing efforts in recycling and waste management, the long-term stability and chemical inertness of common polymers such as polyethylene terephthalate (PET) hinder their degradation and traceability in the environment.^3–5^ Microplastics are even posing a more severe concern due to their health and biological effects, as well as potential hazards.^6,7^ Consequently, the reliable analytical characterization of polymeric materials has become a major scientific and technological challenge, driving extensive research efforts to address this pressing issue.^8–12^ The development of molecular-scale probes capable of detecting, identifying, and quantifying polymeric materials is, therefore, of great scientific and technological interest.

Current detection methods rely mainly on microscopy combined with infrared or Raman spectroscopy and on thermal analysis, which, although chemically informative, are limited by low throughput, matrix interferences, and fluorescence.^12^

In this context, approaches that enable nonoptical detection and quantification of polymers are particularly desirable. Spin labeling provides a complementary strategy by enabling direct detection of plastics through paramagnetic probes and is widely used in biological systems, where nitroxide radicals have become standard reporters of local structure and motion.^13–17^ However, extending such methodologies to synthetic polymers remains challenging, primarily due to difficulties in incorporating stable radicals into inert matrices and maintaining their integrity over extended time scales.^18–21^

Chlorine dioxide (ClO_2_) is a small and stable paramagnetic radical and a powerful oxidant.^22^ Due to its antibacterial and antiviral properties, it is widely used to treat drinking water and employed as an oral disinfectant.^23–26^ Since the ClO_2_ molecule has an unpaired electron residing in a π*-antibonding orbital, it can be detected using electron spin resonance (ESR) spectroscopy.^27–31^ Despite the free radical nature of the molecule, since the electron on the π*-orbital is participating in both oxygen bonds, this resonance hybrid stabilizes the system and is responsible for its relative inertness.^32,33^ Therefore, unlike typical organic spin labels, ClO_2_ can diffuse into polymers without reacting with the host matrix, thus providing a simple route to *in situ* spin labeling.

Indeed, ESR spectroscopy provides an effective approach for characterizing the local environment and dynamics of the spin-label molecules in solids and polymers.^21,34–37^ Furthermore, spin-labeling techniques are widely employed in biological systems, where nitroxide radicals have become standard reporters of local structure and motion.^13–17^ ESR is also known to have superior sensitivity to detect free radicals in the bulk, such as ClO_2_. Given the 1 × 10^9^ spins/G sensitivity of the instrument (as specified by the manufacturer), a quick and conservative estimate yields a limit of detection of <0.1 ppm for ClO_2_. Here, the volume of the solution was estimated to be around 100 μL, and the spectral line width was estimated to be 2 mT. A factor of 1000 was also considered in the molarity to account for the high dielectric losses caused by the water medium.^38^ This is also confirmed by the signal-to-noise ratio of our spectrum for the 30 ppm concentration.

In addition to structural insights, spin labeling can provide a route toward molecular tagging and tracing of plastic materials. The proposed method could complement recently developed fluorescent plastic labeling methods.^39–44^ Such an approach could facilitate the monitoring of degradation processes or the identification of polymer types in mixed waste streams. For this purpose, radicals that can penetrate and remain stable within dense polymer matrices are particularly desirable.

In this work, we demonstrate that ClO_2_ radicals can be efficiently introduced into PET, where they remain stable over a wide temperature range and for sufficiently long times. Temperature-dependent ESR measurements reveal hindered rotational dynamics consistent with confinement within the voids of the polymer strings. The experimental data are accurately reproduced by a modeled spin Hamiltonian. Time-dependent measurements furthermore allowed determination of the diffusion coefficient in the material.

Our findings establish chlorine dioxide as an effective and chemically simple spin label for polymeric materials, opening new perspectives for the ESR-based study and tracing of plastics.

## 2 Methods

### 2.1 Materials

High-purity aqueous solution of chlorine dioxide (ClO_2_) was prepared by *in situ* generation from sodium chlorite, followed by selective permeation of the ClO_2_ gas through a nonporous polymer membrane made of silicone rubber into distilled water. This membrane-permeation method, originally developed by Noszticzius and coworkers,^45^ yields a hyperpure ClO_2_ solution, meaning that the solution is not contaminated with reagents or byproducts because they cannot permeate through the membrane. Such hyperpure solutions are even suitable for biological and physicochemical applications.^26,46–48^

The ClO_2_ concentration in the collected aqueous phase is usually between 3000 and 3500 ppm. Its exact value was determined by iodometric titration,^26^ and it was diluted by distilled water according to the required concentration. All solutions were kept cold, protected from light, and sealed in a bottle to minimize volatilization and decomposition.

Various concentrations of aqueous solutions of ClO_2_, ranging from 30 to 3000 ppm, were prepared and transferred to a 10 mL brown glass vial. A PET film with a lateral size of 1 cm × 4 cm and a thickness of either 12 or 100 μm was immersed in the solution. The vial was sealed and stored in a refrigerator for at least 3 days to achieve a steady state, during which ClO_2_ diffused homogeneously throughout the polymer matrix. The film was removed from the solution immediately before the ESR experiments, and any residual liquid was gently wiped off. A small piece (approximately 5 mm × 5 mm) was then cut and placed into a quartz tube with an internal diameter of 4 mm. When time-dependent measurements were performed, a continuous dry nitrogen flow was applied to remove the chlorine dioxide molecules diffusing out of the PET film. This technique allowed us to determine the diffusion coefficient in PET from the decrease in the ESR intensity.

### 2.2 Electron Spin Resonance

Electron spin resonance (ESR) measurements were carried out using an X-band (∼9.4 GHz) spectrometer operating in continuous-wave mode. The samples were placed in standard quartz tubes with an internal diameter of 4 mm and cooled or heated as required using a variable-temperature unit. Time-dependent measurements were performed in a quartz tube with open ends, and a continuous dry nitrogen flow was applied to remove the chlorine dioxide molecules that diffused out of the PET film. This technique allowed us to determine the diffusion coefficient in PET from the decrease in the ESR intensity. Magnetic field modulation and lock-in detection were employed to record the first derivative of the absorption signal. Here, we wish to note that while the temperature-dependent measurements were acquired in a variable quartz cryostat, the time-dependent measurements were performed without this extension. The dielectric nature of the cryogenic quartz insert lowers the resonance frequency by about 400 MHz (and thus the resonant magnetic field by about 14 mT). These differences are apparent from the comparison of Figures 2 and 3. The *g*-factors and line widths were determined from the field positions and shapes of the resonance lines, following the same procedures as in our previous studies.^49,50^ Calibration of the magnetic field and microwave frequency was performed using 1.5 ppm of Mn:MgO reference with a known *g*-value of 2.0014. The modulation of the magnetic field was 0.1 mT for the concentration- and temperature-dependent measurements. For the time-dependent measurements, a higher 0.5 mT modulation was employed to enable faster accumulation times. No significant distortion of the ESR lines was observed. Microwave powers of 0.2 mW and 0.02 mW were used for the concentration-dependent and temperature-dependent measurements of the pure solutions, respectively. For the 100-μm PET film, a lower microwave power of 2 μW was employed. Time-dependent measurements on the 12-μm PET films were recorded by using 2 mW power. No saturation effects were observed during the measurements. The ESR line width is defined as the half-width at half-maximum, Δ*B* = HWHM, of the spectrum.

### 2.3 Modeling

Theoretical modeling of the ESR spectra was performed using the EasySpin 6 software package^51^ using MATLAB R2024b. Least-squares fitting of parameters was achieved using the Nelder–Mead simplex algorithm, with all fits converging to a tolerance for the error function of 10^–9^. For the aqueous solution, the garlic and for the solid spectra, the pepper functions were used. Above 273 K for the PET samples, the chili function provided the best results. According to the documentation, the garlic function is for isotropic and fast-motional cw EPR spectra of radicals in solution. The pepper function is for solid-state cw EPR spectra for powders, films, and crystals. Lastly, the chili function describes systems in between the previous two, specifically tumbling spin systems in the slow-motional regime.^51^ The details of the spin Hamiltonian used are discussed in Section 3. Relative orientations of the tensors in the molecular frame were neglected during the calculations.

## 3 Results and Discussion

Without any restrictions, an *S* = 1/2 electronic spin interacting with an *I* > 1/2 nuclear spin in a finite ***B*** external magnetic field can be described with the following Hamiltonian:

1 $$H=\mu_{B}BgŜ+hŜAÎ+hÎQÎ-\mu_{n}g_{n}BÎ$$

where the first term is the regular electronic Zeeman interaction between the electron spin, ***Ŝ***, and the external magnetic field, ***B***; the second is the hyperfine term, describing the interaction between the electron and the nuclear spin, ***Î***; and the third term arises from nuclear quadrupolar effects (or the electric field gradient generated by the nucleus). The last term is the nuclear Zeeman term describing the interaction between the nucleus and the magnetic field, whose effect is negligible for ESR experiments. The coupling constants, the *g*-factor, **g**; the hyperfine coupling, **A**; and the quadrupole coupling, **Q**, are rank-three tensors. The Bohr and nuclear magnetons are denoted with μ_B_ and μ_n_, respectively, and *h* is the Planck’s constant.

Since the quadrupole tensor is traceless (e.g., *Q*_*x*_ + *Q*_*y*_ = −*Q*_*z*_), it is common to introduce a nuclear quadrupole coupling constant as *e* ^2^ *Qq*/*h* = 2*I*(2*I* – 1)*Q*_*z*_, and the η = (*Q*_*x*_ – *Q*_*y*_)/*Q*_*z*_ asymmetry parameter, which can take values between 0 and 1. Here, *e* is the elementary charge, *q* is the largest component of the electric field gradient tensor at the nucleus, and *Q* is the electric quadrupole moment of the nucleus.

Considering the case of ClO_2_, there is a single unpaired electron with an *S* = 1/2 spin, which interacts with the chlorine atom that has a nuclear spin of *I* = 3/2. Here, care was taken to note that Cl has two stable isotopes with natural abundances of 75.8% ^35^Cl and 24.2% ^37^Cl with the same nuclear spin. The effect of the two oxygens is neglected, as only ^17^O has a finite nuclear spin of *I* = 5/2, but its abundance is only 0.0367%. Considering the most concentrated sample of 3000 ppm investigated, this would mean a relative ^17^O concentration of as little as ∼1 ppm. Interactions with the protons in water and ice are averaged out and are only considered through the line width. The number of expected peaks to appear in an aqueous solution is thus *N* = 2*I* + 1 = 4 for each Cl isotope. Here, we note that due to the interaction with the solvent media, the observed ESR spectra are strongly solvent- and environment-dependent.^27,52,53^

The ESR spectra of ClO_2_ in aqueous solution with three different concentrations: 30, 300, and 3000 ppm are shown in Figure 1. Low and moderate concentrations of 30 and 300 ppm allow the hyperfine peaks arising from the chlorine atom to be resolved; however, at 3000 ppm, a broadening effect prohibits observation of the four distinct peaks. The observed intensity should be linear with the concentration for highly diluted solutions. This effect is observed for the 30–300 ppm dilutions, where the 10-fold concentration increase results in an almost 10-fold (8-fold) intensity increase. Yet, above this concentration, the dipole interaction between the molecules becomes significant, and the signal broadens, prohibiting the resolution of the individual hyperfine peaks. This means that even after calibration, ESR determination of the concentration is only valid up to ∼300 ppm, as the observed intensity becomes nonlinear above that in the aqueous solution. Here, the intensities are determined by the double integration of the spectra.

**Figure 1:**
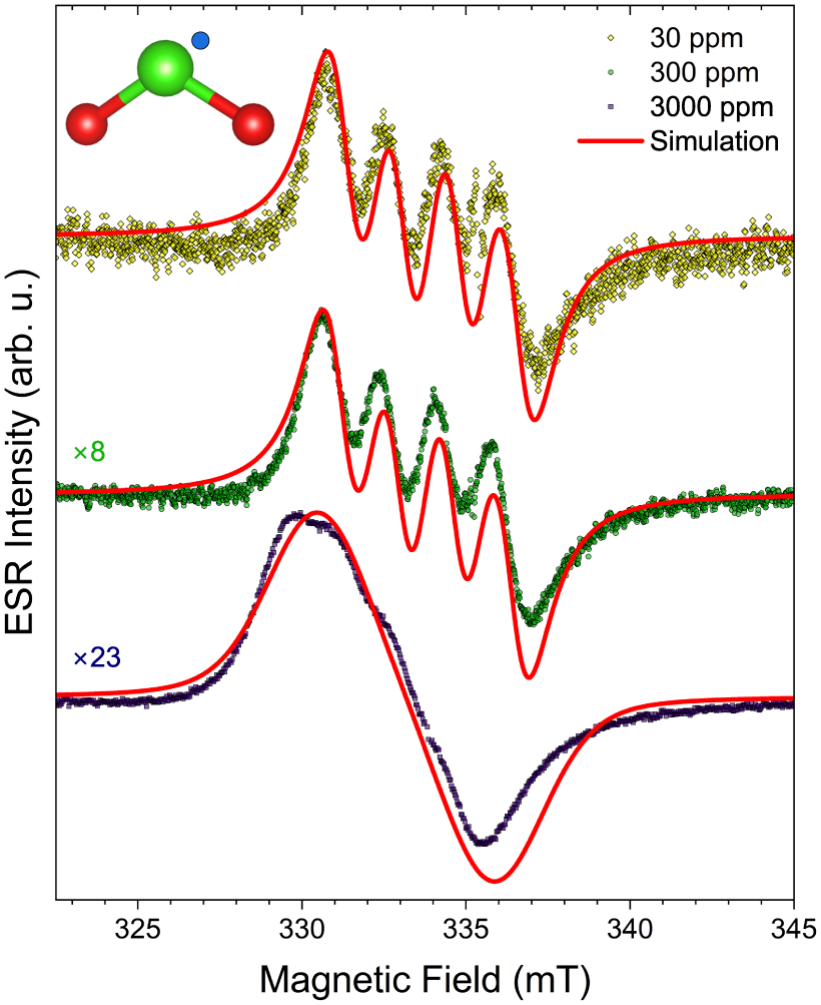
ESR spectra of ClO_2_ in aqueous solution with three different concentrations: 30, 300, and 3000 ppm at 298 K. Multipliers indicate the relative intensity increase, determined by double integration, compared to the 30 ppm solution. While a 10-fold increase in concentration from 30 to 300 ppm increases the signal almost 10-fold, above this threshold, the solution becomes too concentrated, and broadening effects occur due to stronger interactions between the molecules. Solid curves are simulations from the spin-Hamiltonian with parameters fitted to match the experimental data. A detailed analysis is provided in the text.

In the liquid state for the two smaller concentrations, we observe an isotropic *g*-factor of around 2.0093(3), which is expected from the averaging due to the fast motion of the molecules and is in relatively good agreement with the findings of Bennett et al.,^27^ with *g* = 2.010 ± 0.003, and Ozawa et al.^54,55^ with *g* = 2.0106. Interestingly, at the highest concentration, we observed a lower *g*-factor value of *g* = 2.0054(3).

For the hyperfine interaction, we found that a uniaxial coupling of *A*_⊥_ = 65(1) MHz and *A*_∥_ = 19(2) MHz for the 30 and 300 ppm dilutions describes the system best (in terms of reduced χ^2^ values). At low temperatures, in the solid phase, rotational and motional degrees of freedom are completely frozen out, and therefore, only powder averaging occurs. On the other hand, in a solution, motional averaging occurs: the Δω static spectral width of the frozen solution is motionally averaged by the rotational correlation time, τ, when Δωτ ≪ 1. This usually results in a spectrum showing equidistantly split hyperfine lines (the splitting gives the isotropic hyperfine constant). However, the spectrum is very complicated for intermediate values of Δωτ, resulting in a spectrum that is neither a perfect powder distribution nor a perfect motionally averaged type. We unfortunately have no additional information about the expected molecular rotational correlation time of ClO_2_; thus, we cannot independently verify which regime is encountered herein. At the same time, Bennett et al.^27^ found an isotropic coupling of *A* = 48 MHz (albeit their spectral resolution was limited at that time), which can be interpreted as a weighted average of our values, *A*_iso_ = (2*A*_⊥_ + *A*_∥_)/3 ≈ 50(1) MHz. Using an isotropic model, we obtained *A*_iso_ = 49(1) MHz, in agreement with the average and the literature value. Oddly, at 3000 ppm concentration, the observed hyperfine coupling becomes nearly isotropic with values of *A*_⊥_ = 42(1) MHz and *A*_∥_ = 32(2) MHz (or 39(1) MHz in the isotropic model).

Upon cooling below the freezing point of water, a considerable broadening of the ESR line is observed, as shown in Figure 2a and f. This is in contrast to other—organic^52^ and inorganic^53^—solvents, where cooling narrows the line. Here, this broadening is only observed below 220 K. The line is also inhomogeneously broadened, which is considered through a Gaussian component of the line width. In the solid state, most of the parameters differ from those observed in the liquid phase, as interactions are no longer averaged out. In water ice, the *g*-factor becomes uniaxial, while the hyperfine coupling has three distinct directions, as presented in Figure 2b and c, respectively. A strong but isotropic quadrupolar coupling is also observed, in agreement with previous measurements on ClO_2_ trapped in inert matrices at low temperatures.^29^ Nonetheless, most spectral parameters show little temperature dependence in the observed temperature region. Notably, the anisotropies of the *g*-factor, the hyperfine coupling, and the quadrupole coupling exhibit a weak maximum between 220 and 250 K.

**Figure 2:**
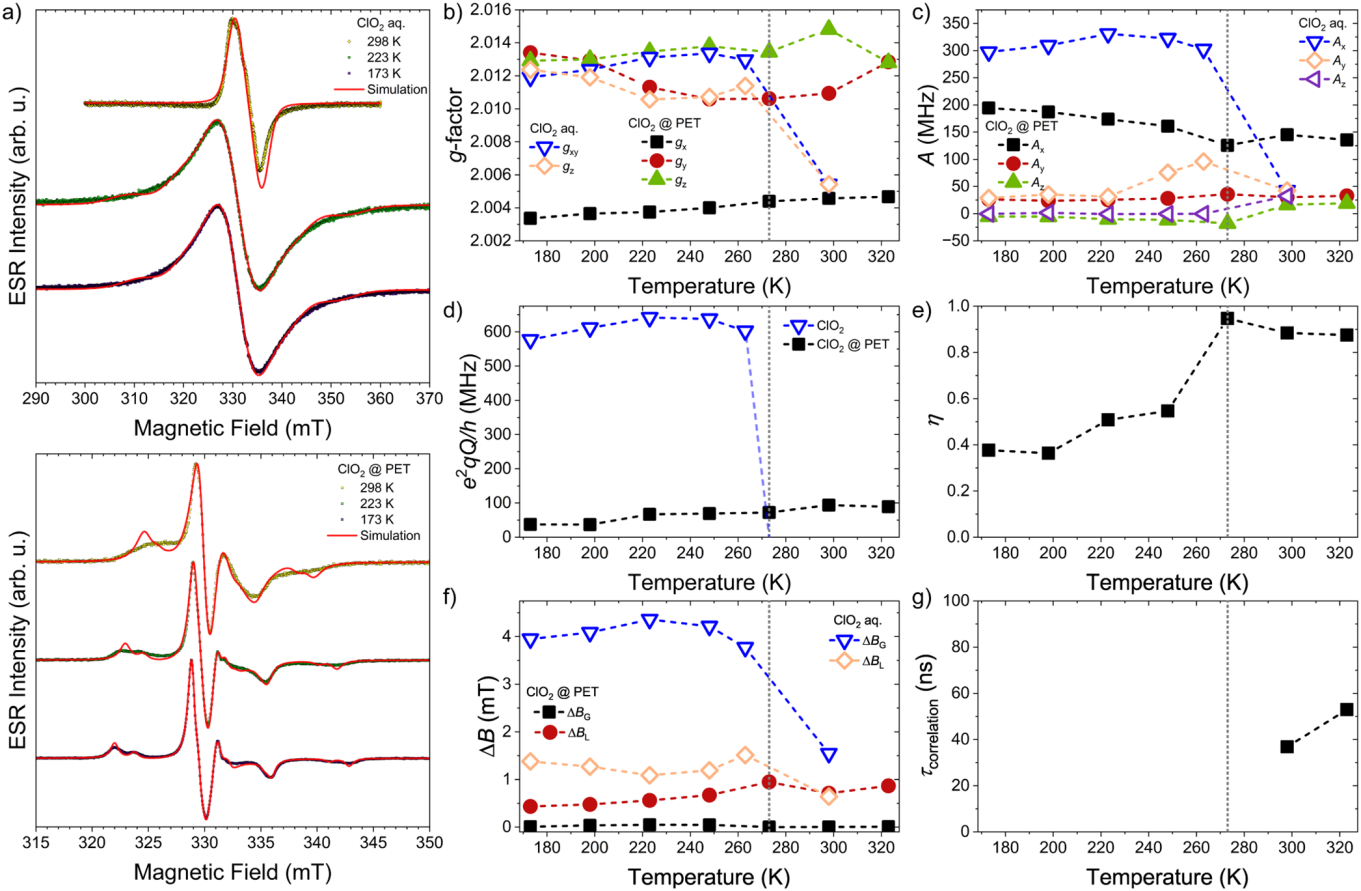
a) Detected derivative cw ESR spectra of ClO_2_ in a 3000 ppm aqueous solution at 298, 223, and 173 K (top) and the same for a 100-μm-thick PET sample soaked in the solution for days. Please note the drastic change in the line shape depending on the solid matrix. Solid lines are simulations with parameters given in panels b to g. The details of the spin model can be found in the main text. b-g) Parameters obtained from the spin model: *g*-factors, hyperfine couplings (*A*), nuclear quadrupole coupling constant (*e* ^2^ *qQ*/*h*) and its asymmetry parameter (η), line widths (Δ*B*), and rotational correlation time (τ_correlation_), respectively. In the solid phase, all parameters show little variation with the temperature. On the other hand, around the phase transition of water, they do change drastically. Moreover, note the significant difference between the aqueous solution and when the molecules are embedded in the PET matrix. Vertical dashed lines denote the melting point of water under ambient pressure. The semitransparent dashed blue line in panel d suggests that the quadrupolar coupling averages to zero at 273 K in the aqueous ClO_2_.

When introduced into PET, all spectral observables become rhombic both below and above the freezing point of water. This indicates that even in a liquid solution state, the polymer matrix prevents certain motions and interactions from being partially averaged out. A similar behavior is observed in KClO_4_,^56^ synthetic zeolites,^28^ and on the surface of MgO,^57^ where the crystal symmetry strongly affects the coupling constants. Compared with the water-ice matrix, the anisotropy of the *g*-factor is much more pronounced for the *g*_*x*_ direction (Figure 2b). The magnitude of the hyperfine coupling, as well as its asymmetry, is moderately reduced; in turn, the quadrupole coupling is reduced by 1 order of magnitude, as shown in Figure 2c and d. The latter also displays a strong anisotropy with η values close to 1 above the freezing point and around 0.5 below (Figure 2e). Furthermore, the observed line width is significantly narrower, and almost no Gaussian broadening is observed (Figure 2f). Most parameters, except the anisotropy of the quadrupole coupling, have a slight temperature dependence again. Above the melting point of water, neither the solid nor the fast-moving liquid model adequately describes the system. Instead, a slowly moving, tumbling description was found to be appropriate, where a rotational correlation parameter was introduced and is on the order of 50 ns, as shown in Figure 2g. Another intriguing observation is that even though the observed intensity as a function of concentration in the aqueous solution starts to deviate from a linear relation above 300 ppm, the PET matrix seems to alleviate this limitation, as the ClO_2_ molecules are more separated, allowing reliable intensity determination in PET films soaked in up to 3000 ppm aqueous solutions of ClO_2_.

The fit quality of ClO_2_ embedded in the PET is reasonably good at low temperatures (e.g., at 173 K); however, it is apparently inferior at room temperature. This is most probably due to an ill-defined rotational axis and rotational correlation time, as well as the possible presence of a distribution in the latter. This may arise from the nonuniform spacing/voids of the PET matrix. At low temperatures, the fully static, randomly oriented powder distribution of the molecules is a well-defined physical state. In contrast, a partially hindered rotation with a distributed correlation time cannot be uniquely simulated.

Lastly, we study the kinetics of how ClO_2_ diffuses out from the PET. The previously soaked samples were placed in a continuous flow of dry nitrogen gas to remove the outgassed molecules, and a time series of ESR spectra was accumulated, as presented in Figure 3a. Accumulation of a single spectrum took about 42 s, and the time difference between the accumulations was 5 min. Here, it must be noted that the preparation of the measurement required some time (e.g., transferring the sample and adjusting the gas flow). This time was measured and is considered as a finite shift along the time axis.

**Figure 3:**
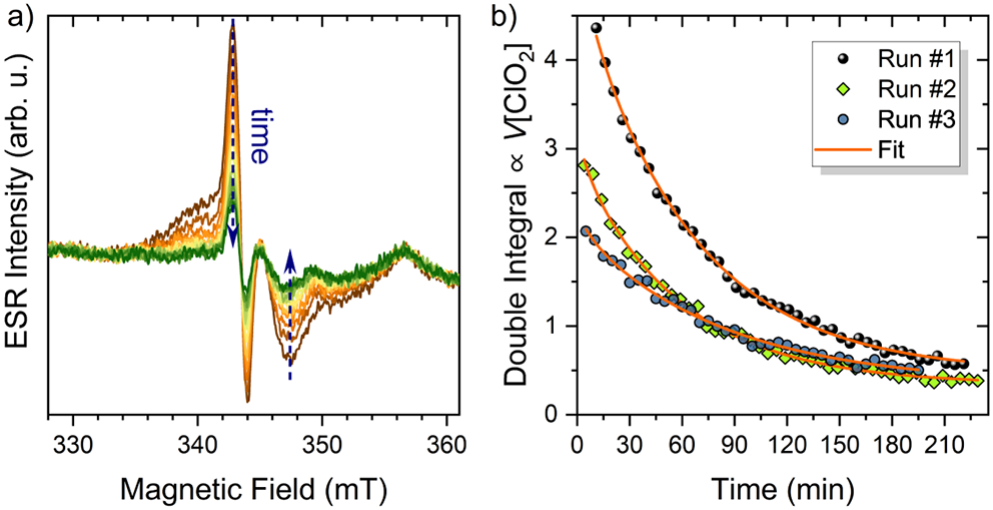
a) A time series of ESR measurements performed on a 12-μm-thick PET film, initially soaked in a 2900 ppm aqueous solution of ClO_2_. All measurements were performed at room temperature. The feature at 357 mT arises from the sample holder and was excluded from later evaluation steps. The time difference between two adjacent spectra is 5 min. Please note the systematic decrease in the spectral intensity. b) Time dependence of the ESR intensity from double integration of the spectrum for three separate runs. The curves follow an exponential decay in time, as dictated by the outdiffusion and removal of the ClO_2_ molecules from the PET film. The orange curve is a fit based on the theoretical discussion presented in the main text. Please note that the decay characteristic is independent of the initial settings. The obtained diffusion coefficient is *D* = (3.91 ± 0.74) × 10^–15^ m^2^/s at room temperature.

The decrease in the ESR signal can be modeled as a diffusion process as the ClO_2_ molecules leave the PET film. Using Fick’s laws, the diffusion equation for the *c*(***r***, *t*) concentration in the film reads:

2 $$\frac{\partial c\left(r,t\right)}{\partial t}=D\nabla^{2}c\left(r,t\right)$$

where *D* is the diffusion coefficient, assumed to have no spatial dependence. The obtained ESR intensity is directly proportional to the total amount of substance—and hence the integral of the concentration—as there are no other spin species present in the system, *I*_ESR_ ∝ ∫_*V*_ *c* = *V*[ClO_2_]. Denoting the surface of the cross-section of the film with *A*, and the thickness with *L*, the bulk volume, measured by the ESR, is *V* = *A* × *L*. As we have no spatial resolution, the concentration has to be integrated for the whole bulk at every *t*. Since the thickness of the film is considerably smaller, most molecules will leave the film through the faces with the *A* area. This simplifies the mathematical problem to a single spatial dimension, *x*, which is taken along the thickness of the film. This also means that isoconcentration surfaces are planes parallel to the *A* area. Before the measurement, the samples were soaked in the ClO_2_ solution sufficiently long to reach a steady state, meaning that the initial concentration of ClO_2_ was homogeneous in the film: *c*(*x*, *t* = 0) = *c*_0_. Now, it is safe to assume that the concentration on the surface is constantly zero, *c*(0) = *c*(*L*) = 0, as the nitrogen flow removes any outgassed molecules. The diffusion equation can be analytically solved for this case (homogeneously filled infinite plane-parallel film) with the given boundary conditions. The solution can be obtained via the method of separation of variables and then using a Fourier series expansion; details of the derivation can be found in the Supporting Information and in ref 58. The resulting infinite series reads:

$$c\left(x,t\right)=\frac{4c_{0}}{\pi }\overset{\infty }{\underset{j=0}{\sum }}\left\{\frac{1}{2j+1}\sin\left[\left(2j+1\right)\pi \frac{x}{L}\right]\;\times \exp\left[-{\left(\frac{\left(2j+1\right)\pi }{L}\right)}^{2}Dt\right]\right\}$$ 3

The ESR intensity is assumed to be a linear function of the concentration integrated over the whole bulk, with an added background (*I*_0_). Because the concentration changes considerably only through the thickness of the film, the integral needs to be taken only over the thickness, and then it needs to be multiplied by the cross-section of the film. The resulting formula for the intensity is:

4 $$I_{\mathrm{ESR}}\left(t\right)=\mathrm{\eta ̃}A\int_{0}^{L}c\left(x,t\right)dx+I_{0}=\mathrm{\eta ̃}A\frac{8Lc_{0}}{\pi^{2}}\overset{\infty }{\underset{j=0}{\sum }}\left\{\frac{1}{{\left(2j+1\right)}^{2}}\times \exp\left[-{\left(\frac{\left(2j+1\right)\pi }{L}\right)}^{2}Dt\right]\right\}+I_{0}$$

Here, η̃ and *I*_0_ are instrumental parameters. Keeping only the first two terms of the series (*j* = 0, 1), the expression already satisfactorily fits the obtained intensities, as presented in Figure 3b. The diffusion coefficient obtained from the measurement of ClO_2_ in PET is *D* = (3.91 ± 0.74) × 10^–15^ m^2^/s at room temperature.

Beyond providing microscopic insight into radical dynamics in PET, this approach has direct implications for environmental polymer tracking. Spin labeling with ClO_2_ enables the detection of plastics using ESR spectroscopy, which is inherently insensitive to optical opacity, fluorescence background, or complex chemical matrices. This makes the method particularly attractive for environmental samples, such as drinking water, sediments, soils, or wastewater sludges, where conventional optical and thermal techniques often fail. Moreover, the sensitivity of ESR spectral parameters to the local polymer environment suggests that different polymer classes may yield distinguishable spectral signatures, enabling polymer-specific identification.

## 4 Conclusions

In conclusion, we have demonstrated that chlorine dioxide could serve as an efficient, stable, and inorganic spin label for polyethylene terephthalate (PET). Given the similarity in chemical properties, ClO_2_ might be used for other types of polymers as well, aiding further studies. By exposing PET to ClO_2_, radicals become incorporated into the polymer matrix, where they remain ESR-active over a wide temperature range and for extended periods of time. Since PET (and most plastics in general) has low microwave absorption, there is no practical limit to the thickness in labeling efficiency. Only the finite time required by the diffusion of the ClO_2_ molecules has to be considered to achieve a homogeneous distribution in the plastic. On the other hand, there is a technical limitation due to the instrumentation in which the sample has to be placed inside a microwave resonator that has a finite active volume of about a few cm^3^, typically. Temperature-dependent ESR spectroscopy revealed restricted rotational dynamics indicative of hindered molecular motion within the voids of the polymer. The recorded spectra were accurately described by a fitted spin Hamiltonian, reflecting the nature of the environment in the radical’s intrinsic electronic properties. Furthermore, time-dependent measurements enabled the determination of the diffusion coefficients of the molecules in the plastic.

These findings establish a simple and general approach to spin labeling otherwise inert plastics without covalent modification or external additives during the manufacturing process. Beyond their use as local probes, embedded ClO_2_ radicals could provide a platform for the identification, tracing, or degradation monitoring of polymeric waste, polymer-based currencies, and printed circuit boards (PCBs). From an environmental perspective, the demonstrated ClO_2_-based spin labeling provides a complementary route to existing micro- and nanoplastic detection techniques.^39–44^ Unlike infrared- or Raman-based methods, ESR detection does not rely on optical properties and can operate in chemically and structurally complex environments. The proportionality between ESR signal intensity and radical concentration further enables the quantitative assessment of polymer content. Time-dependent measurements offer access to diffusion and release kinetics that are relevant for studying polymer aging, transport, and degradation. As such, this approach has the potential to contribute to future strategies for tracing, quantifying, and differentiating polymeric materials in environmental samples, addressing several key challenges highlighted in the Introduction.

The presented method thus opens new perspectives for both fundamental studies of polymer physics and practical applications in sustainable materials research and anticounterfeiting.

## Supplementary Material



## Notes

Contains the derivation of the time- and space-dependent concentration in the PET film described by eq 3 (PDF)
