# Supplementary material for: Transient Spin Labeling of Plastics with Chlorine Dioxide
Source: ACS Phys Chem Au. 2026 Mar 4;6(2):348–55. doi: 10.1021/acsphyschemau.5c00140 (PMC13022717; doi:10.1021/acsphyschemau.5c00140)
Supplement: Supplementary file 1 [file pg5c00140_si_001.pdf]

Transient Plastic Spin Labeling with Chlorine Dioxide

Bence G. Márkus[†,1,*] Sándor Kollarics,[2,3,†] Kristóf Kály-Kullai,[2] Bernadett Juhász,[2]
Dávid Beke,[3,4] László Forró,[1] Zoltán Noszticzius,[2] and Ferenc Simon[2,3,*]
[1]*Stavropoulos Center for Complex Quantum Matter, Department of Physics and Astronomy, University of Notre Dame, Notre Dame, Indiana 46556, USA*
[2]*Department of Physics, Institute of Physics, Budapest University of Technology and Economics, Műegyetem rkp. 3., H-1111 Budapest, Hungary*
[3]*Institute for Solid State Physics and Optics, HUN-REN Wigner Research Centre for Physics, PO. Box 49, H-1525, Hungary*
[4]*Kandó Kálmán Faculty of Electrical Engineering, Óbuda University, Tavaszmező u. 17., H-1084 Budapest, Hungary*

SUPPORTING INFORMATION

This Supplementary Information contains the derivation of the time- and space-dependent concentration in the PET film described by Eq. (3) of the main text.

Derivation of the Time and Space Dependent Concentration of a Compound Diffusing out of an Infinite Plan-Parallel Medium

We assume that our compound is nonreactive and that its transport is governed solely by diffusion. Thus, the time evolution of its concentration is described by the diffusion equation:

$$\frac{\partial c(\boldsymbol{r},t)}{\partial t} = D\nabla^2 c(\boldsymbol{r},t), \tag{S1}$$

where t denotes time, $\boldsymbol{r}$ the space vector, $c(\boldsymbol{r},t)$ the concentration of the compound, D the diffusion coefficient. The medium is assumed to be homogeneous; therefore, D is constant and independent of space and time.

We consider the medium from which c diffuses out to be an infinite plan-parallel volume. Outside the medium, a well-mixed gas phase is assumed. Therefore, the concentration is 0 there. The bounding surfaces of the medium are taken to be the planes at $x = 0$ and $x = L$. Furthermore, we assume that at the initial time ($t = 0$) the concentration is spatially uniform within the medium and equal to c_0 for $0 < x < L$.

In such an arrangement c does not depend on y or z, thus Eq. (S1) reduces to a one-dimensional diffusion equation:

$$\frac{\partial c(x,t)}{\partial t} = D\frac{\partial^2 c(x,t)}{\partial x^2}. \tag{S2}$$

The initial condition is therefore $c(x,0) = c_0$ for $0 < x < L$, and $c(x,0) = 0$ elsewhere. The boundary conditions are $c(0,t) = c(L,t) = 0$.

The solution can be obtained via the separation of variables method. Meaning that we try to find a solution where c is the product of a space-dependent (and time-independent) function and a time-dependent (and space-independent) function, so that

$$c(x,t) = X(x)\cdot T(t). \tag{S3}$$

By substituting Eq. (S3) into Eq. (S2) and dividing both sides of the equation by $c(x,t)$ we obtain

$$\frac{\mathrm{d}T(t)/\mathrm{d}t}{T(t)} = D\frac{\mathrm{d}^2X(x)/\mathrm{d}x^2}{X(x)}. \tag{S4}$$

Here, the left side depends solely on time, the right side solely on x. The equation can hold for arbitrary time and x values only if both sides are equal with the same constant, which we denote by K:

$$\frac{\mathrm{d}T(t)/\mathrm{d}t}{T(t)} = D\frac{\mathrm{d}^2X(x)/\mathrm{d}x^2}{X(x)} = K. \tag{S5}$$

This way, we have two ordinary differential equations to solve. We start with the time-dependent part; it can be reformulated as:

$$\frac{\mathrm{d}T(t)}{\mathrm{d}t} = KT(t), \tag{S6}$$

which is a linear differential equation. Its solution is $T(t) = \exp(Kt)$, where the multiplicative integration constant is omitted at this stage and will be discussed later.

As the concentration cannot grow infinitely, the constant K must be negative. We therefore introduce the so-called time constant (or characteristic time): $\tau = -1/K$. With them, the solution for the time-dependent part is formulated as

$$T(t) = \mathrm{e}^{-t/\tau}, \tag{S7}$$

where $\tau > 0$ must hold.

Next, we look at the space-dependent function. Our differential equation to solve is the following:

$$\frac{\mathrm{d}^2X(x)}{\mathrm{d}x^2} = -\frac{1}{\tau D}X(x). \tag{S8}$$

Furthermore, $X(x)$ must satisfy the same boundary conditions as $c(x,t)$, that is $X(0) = X(L) = 0$.

The general solution has the form $X(x) = A\sin(kx) + B\cos(kx)$. Due to the boundary condition of $X(0) = 0$, B must be zero. From the other boundary condition, it follows

* Corresponding author: bmarkus@nd.edu
† These authors contributed equally.
* Corresponding author: simon.ferenc@ttk.bme.hu

that $kL = n\pi$ must hold, where n is an integer. Furthermore, due to the symmetry of the problem, the solution must remain invariant upon exchanging the two boundaries. This excludes even multiples of π, and therefore $kL = (2j+1)\pi$ must hold, where j is an integer. The allowed values for k are

$$k_j = \frac{(2j+1)\pi}{L}. \tag{S9}$$

Substituting these expressions into Eq. (S8) yields

$$-k_j^2 A_j \sin(k_j x) = -\frac{1}{\tau_j D} A_j \sin(k_j x), \tag{S10}$$

from which the corresponding time constants can be deduced:

$$\tau_j = \frac{1}{k_j^2 D} = \left(\frac{L}{(2j+1)\pi}\right)^2 \frac{1}{D} \tag{S11}$$

The diffusion equation gives infinitely many solutions, and the complete solution for c is given by their linear combination, which is an infinite sum in the following form:

$$c(x,t) = \sum_{j=0}^{\infty} A_j \sin(k_j x)\, \mathrm{e}^{-k_j^2 D t}. \tag{S12}$$

This sum should contain linearly independent terms; therefore, negative values for j were excluded (due to symmetry reasons, $\sin(k_{-j}x) = -\sin(k_{j-1}x)$).

The initial condition can be used to determine the coefficients A_j. Setting $t = 0$ in Eq. (S12) yields

$$c(x,0) = \sum_{j=0}^{\infty} A_j \sin(k_j x) = c_0. \tag{S13}$$

This shows that the coefficients, A_j, are the Fourier-coefficients of the initial value.

To calculate these, both sides are multiplied by $\sin(k_i x)$ and integrated over the domain:

$$\int_0^L \left\{ \sin(k_i x) \sum_{j=0}^{\infty} A_j \sin(k_j x) \right\} \mathrm{d}x = \int_0^L c_0 \sin(k_i x)\, \mathrm{d}x. \tag{S14}$$

Interchanging the order of summation and integration (as both A_j and k_j are independent of x) leads to

$$\sum_{j=0}^{\infty} \left\{ A_j \int_0^L \sin(k_j x) \sin(k_i x)\, \mathrm{d}x \right\} = c_0 \int_0^L \sin(k_i x)\, \mathrm{d}x. \tag{S15}$$

We remark that due to Eq. (S9), $\sin(k_j L) = 0$, and $\cos(k_j L) = -1$. This allows the right-hand side to be evaluated as:

$$c_0 \int_0^L \sin(k_i x)\, \mathrm{d}x = -c_0 \left.\frac{\cos(k_i x)}{k_i}\right|_0^L = \frac{2c_0}{k_i}. \tag{S16}$$

By using the product-to-sum trigonometric identity, the integral on the left-hand side can be reformulated as follows:

$$\int_0^L \sin(k_j x)\sin(k_i x)\, \mathrm{d}x = \\ = \frac{1}{2}\int_0^L \cos[(k_j - k_i)x]\, \mathrm{d}x - \frac{1}{2}\int_0^L \cos[(k_j + k_i)x]\, \mathrm{d}x. \tag{S17}$$

For $i \neq j$, both $(k_j - k_i)L$ and $(k_j + k_i)L$ are even multiples of π, thus both integrals are taken over whole periods, meaning they are 0. In the case of $i = j$, this is only true for the second integral, $\cos[(k_i - k_i)x] = 1$, thus the first term evaluates as $L/2$. Substituting all of these integral values to Eq. (S15) yields

$$A_i \frac{L}{2} = \frac{2c_0}{k_i}. \tag{S18}$$

Reordering for A_i, relabeling the index i to j, and using Eq. (S9) gives

$$A_j = \frac{4c_0}{k_j L} = \frac{4c_0}{(2j+1)\pi}. \tag{S19}$$

Finally, substituting Eqs. (S9) and (S19) into (S12) gives the complete time- and space-dependent solution of Eq. (S2):

$$c(x,t) = \frac{4c_0}{\pi} \sum_{j=0}^{\infty} \left\{ \frac{1}{2j+1} \sin\left[(2j+1)\pi\frac{x}{L}\right] \times \\ \exp\left[-\left(\frac{(2j+1)\pi}{L}\right)^2 Dt\right] \right\}. \tag{S20}$$
